# Comparing the effectiveness of a medication knowledge base product as designed with real-world hospital implementations using the Leapfrog Group’s Computerized Physician Order Entry (CPOE/EHR) Evaluation Tool

**DOI:** 10.1093/jamiaopen/ooaf176

**Published:** 2026-01-06

**Authors:** Zoe Co, Howard R Strasberg, Bruce R Hanway, Dean F Sittig, David C Classen

**Affiliations:** Department of Learning Health Sciences, University of Michigan Medical School, Ann Arbor, MI 48104, United States; Department of General Internal Medicine, Brigham and Women’s Hospital, Boston, MA 02120, United States; Clinical Effectiveness, Wolters Kluwer Health, Indianapolis, IN 46240, United States; Clinical Effectiveness, Wolters Kluwer Health, Indianapolis, IN 46240, United States; Department of Clinical and Health Informatics, McWilliams School of Biomedical Informatics, University of Texas Health Science Center at Houston, Houston, TX 77030, United States; Informatics Review LLC, Lake Oswego, OR 97034, United States; Division of Epidemiology, University of Utah School of Medicine, Salt Lake City, UT 84132, United States

**Keywords:** patient safety, clinical decision support systems, medication safety

## Abstract

**Objectives:**

We sought to compare and analyze the performance of a medication knowledge base (MKB) vendor’s (Medi-Span) fully enabled, factory settings product to operational implementations in hospitals using the Leapfrog Group’s Computerized Physician Order Entry (CPOE)/electronic health record (EHR) Evaluation Tool.

**Materials and Methods:**

We randomly selected a hospital that used the MKB vendor’s product to complete the Leapfrog CPOE/EHR Evaluation Tool in 2018 and used this version of the tool to assess the MKB vendor’s fully enabled product. Next, we retrospectively evaluated all hospitals, regardless of EHR vendor, that used the MKB vendor’s product in the tool from 2017 to 2019. We fit a multivariate linear regression model with hospitals’ overall scores as the dependent variable and EHR vendor as the independent variable.

**Results:**

The fully enabled version of the MKB vendor achieved a 94% overall score in the tool. The mean overall score across 358 hospitals from 2017 to 2019 ranged from 61.1% to 68.8%. Finally, the regression model revealed EHR Vendor B hospitals scored 3.6 percentage points lower (*P* = .01) than EHR Vendor A hospitals, but the model only explained 3.7% of the variation in overall scores.

**Discussion:**

The study revealed variations in the effectiveness of implementation and configuration of the MKB vendor’s product across all EHR vendors at the hospital level.

**Conclusion:**

Our regression model found that EHR vendor had minimal effect on the overall tool scores. Therefore, EHR vendor is not nearly as important in the performance on the CPOE/EHR Evaluation Tool as the choices made in configuring the MKB vendor’s product within a specific EHR.

## Background and significance

The Health Information Technology for Economic and Clinical Health (HITECH) Act of 2009 distributed more than $30 billion in incentives to hospitals, clinics, and physicians for the adoption of electronic health record (EHR) systems in the United States.^1^ It was widely successful in terms of adoption of EHR systems, with more than 95% of hospitals and almost 90% of clinics having implemented them.^1^^,^^2^ A major part of HITECH was the Meaningful Use requirements, which healthcare systems needed to meet to receive the incentive payments.^3^ The Meaningful Use program included requirements for using EHR systems for recording specific patient data, integrating clinical decision support (CDS) tools into clinical care, using health information exchange, and reporting quality improvement data.^3^ In conjunction with healthcare systems, EHR vendors also had to get their EHR products Meaningful Use certified. This was done mainly by the Office of the National Coordinator, now known as the Assistant Secretary for Technology Policy through authorized testing and certification bodies that reviewed each vendor’s products preimplementation.^4^ Although there was a certification requirement for real-world testing of these vendor products, the postimplementation evaluations that were part of the program were never effective.^3^

### Using medication knowledge bases for medication-related clinical decision support

In conjunction with implementing EHR systems, healthcare systems often chose to implement medication knowledge bases as part of their decision support tools to meet the Meaningful Use requirements. These knowledge bases are an important component of CDS tools, as they contain the rules and logic from which these tools’ alerts are triggered.^5^ Therefore, most hospitals installed a third party commercial medication knowledge base (MKB) to guide their medication decision support within their operational EHR vendor product.

### Implementation of these knowledge bases and local configuration of EHRs

As hospitals started implementing EHR systems, they configured their EHR products to fit local needs. One common configuration that healthcare systems made to their EHR systems was to their medication-related CDS tools. Specifically, healthcare systems could decide which alerts to use and not to use. These configuration decisions were not assessed in the EHR vendor certification programs because of obvious logistical challenges in testing every hospital’s individual EHR implementation. Thus in 2009, experts at Brigham and Women’s Hospital (Boston, MA) and the University of Utah (Salt Lake City, UT) released the Computerized Physician Order Entry (CPOE)/EHR Evaluation Tool, which is currently administered by the Leapfrog Group.^6^ A key component of this tool is that it evaluates the performance of medication decision support interventions as configured and operationalized within a local hospital’s EHR system.

### Using the CPOE/EHR Evaluation Tool to evaluate EHR configuration

Since 2009, annual hospital participation using the CPOE/EHR Evaluation Tool to assess their EHR functionality has grown from a handful of hospitals to almost 3000 hospitals using the test in 2024. The tool currently consists of 8 order checking categories that assess basic decision support alerts like drug-route alerts to advanced decision support alerts like drug-diagnosis alerts.^7^ Hospitals take the test autonomously, where they are provided with 10-14 fictitious test patients that they enter as an inpatient into their production EHR system.^8^ Each test patient includes demographic details such as age, weight, allergies, diagnosis, and relevant laboratory values. Next, a set of associated medication test orders are provided to hospitals, where licensed prescribers record any advice or information they receive as they enter these test orders into their EHR system. After they are finished, hospitals receive immediate feedback in the form of an overall percentage score of unsafe medication orders detected, as well as individual order category scores. These scores are calculated by taking the number of correctly alerted test orders divided by the total number of orderable test orders. Scores for all test categories as well as an overall test score are recorded for each organization that takes the test along with several other descriptive organizational characteristics (eg, name of organization, EHR vendor used, medication knowledge base used, and other descriptors). These scores are available for all organizations that took the test between 2017 and 2023 apart from 2020 and 2021 when the test was not required due to the COVID pandemic.

Multiple studies have evaluated the results of the CPOE/EHR Evaluation Tool.^9–13^ These studies revealed that hospital performance in the tool has generally improved over time, with recent overall test scores demonstrating approximately 75% overall compliance with basic CDS best practices, and continued lower adherence to best practices in advanced decision support areas.^11^^,^^12^ Examples of the latter area include failing to intercept medication orders that would harm a fetus, medication orders that need adjustment for renal dysfunction, and medication orders that might overdose a patient based on recent drug levels.^11^ Also, no correlation with scores in the tool could be found with meaningful use of specific EHR vendors yet test results could be correlated with hospital performance on other publicly reported quality measures.^11^^,^  ^14^ Further analysis of these studies continually demonstrates that much more variation in test results occurred within hospital groups with the same EHR vendor rather than across hospitals with different EHR vendors. In these previous studies, the focus was on stratifying test results based on the EHR vendor, regardless of the knowledge base vendor these hospitals used. Since hospitals can customize both their EHR systems and their knowledge bases, it is important to expand analyses of the CPOE/EHR Evaluation Tool to the level of the MKB vendors.

### Objectives

In this study, we sought to compare and analyze the performance of a MKB vendor’s fully enabled, factory settings product to an operational implementations at hospitals using the Leapfrog Group’s CPOE/EHR Evaluation Tool. Our 3 research questions were: (1) What is the difference in performance in the CPOE/EHR Evaluation Tool between a MKB vendor’s fully enabled, factory settings product (Medi-Span, Wolters Kluwer Health, Indianapolis, IN, United States) and the same MKB vendor’s product as implemented by hospitals? (2) What is the variation in performance of hospitals in the CPOE/EHR Evaluation Tool using the same MKB as implemented and configured within several commercially available EHRs, and (3) Is there an association between EHR vendor and overall test scores in the CPOE/EHR Evaluation Tool among the hospitals that use the same MKB?

## Materials and methods

### Evaluation of the medication database vendor’s fully enabled factory settings product

Much like the Meaningful Use certification process, we began by assessing the MKB vendor’s fully enabled factory settings product with the CPOE/EHR Evaluation Tool. Medi-Span is a medication knowledge base that offers both embedded drug data and an automated clinical screening solution. It is designed to alert clinicians and pharmacists making prescribing and dispensing decisions about avoidable medication errors, inappropriate dosing, and potential adverse events. Clinical screening modules include drug-drug interactions; drug-allergy screening; drug-gene interactions; dose screening; drug-disease contraindications; duplicate therapy screening; route contraindications; and pregnancy, lactation, age, and gender screening. In their fully enabled, factory settings product, all possible alerts are displayed, and this is the version of the MKB vendor’s product that we used for the comparison. From this point onward, we will refer to this product as the “fully enabled product.”

Next, we randomly selected a test that a hospital took using the CPOE/EHR Evaluation Tool and this MKB vendor’s product in 2018. We used this exact version of the tool for the evaluation of the MKB vendor’s fully enabled product. Note, to prevent gaming of the test, the Leapfrog Group randomly generates a slightly different, but equally challenging, test for every hospital from a larger set of test questions. For the evaluation itself, we worked with the MKB vendor to assess a live version of their fully enabled product before it was implemented within an EHR using a “test harness.” This was done to compare the performance of the MKB vendor’s fully enabled product before implementation at hospitals (in vitro) to actual operational implementations at hospitals with potential local customizations (in vivo).

The version of the CPOE/EHR Evaluation Tool that was used for this assessment included 10 order checking categories and 1 subcategory, fatal orders. Starting in 2020, the CPOE/EHR Evaluation Tool underwent changes to its content and scoring algorithm as well as a COVID-related pause in testing in 2020 and 2021. Since we wanted to compare performance across years, we decided to use the 2017-2019 version of the tool, as there were minimal changes to the tool over this time. In comparison to the current version of the CPOE/EHR Evaluation Tool, the content for most of the order categories did not change; rather the organization of the tool did. Specifically, the drug-drug interaction and therapeutic duplication order categories are now combined into one category, “Inappropriate Medication Combinations,” and the drug dose (daily) and the drug dose (single) order categories are combined into one category, “Excessive Dosing.” In addition, the tool no longer assesses for drug allergy alerts, as hospitals consistently performed well in this area since 2018.^11^ Next, fatal orders in the tool were medication orders that could cause a fatality if administered to a patient. Lastly, the drug level monitoring and drug laboratory interaction test orders were not included in this evaluation, as these types of decision support could not be tested by the MKB vendor.

### Statistical analysis from national sample

We analyzed test results from the 2017-2019 version of the Leapfrog CPOE/EHR Evaluation Tool from hospitals which used the MKB vendor as implemented within several different commercially available EHR systems. Since these test results included drug laboratory and drug monitoring order category scores, we recalculated all hospitals’ scores to exclude these order categories. Using these new overall scores, we calculated descriptive statistics including the mean scores, SD, median, minimum, and maximum scores. Next, we created a dot plot of the overall test scores stratified by EHR vendor to illustrate the range of overall test scores within each EHR vendor. We also created line plots to show the change in mean overall scores across hospitals over the 3-year testing period. This was also done for each order category score across all hospitals. For the fatal orders, we created bar graphs to show the percentage of fatal orders hospitals appropriately detected across all 3 years.

Finally, we created a multivariate linear regression model with hospitals’ overall scores as the dependent variable and EHR vendor as our primary independent variable of interest. The EHR vendors these hospitals used are among the leading EHR vendors by market share. For this model, we combined all 3 years of data. We also included hospital demographics as control variables, which we obtained by linking our dataset with the American Hospital Association’s Annual Hospital Survey.^15^ The hospital demographics we selected included healthcare system membership (part of healthcare system or not), ownership (nonprofit, for-profit or government-owned), hospital size (small, medium, or large), location (urban vs rural), and teaching hospital status. The model also included year-fixed effects to account for any time-related factors and we calculated robust SEs clustered at the hospital and EHR vendor levels. All analyses were conducted using R and STATA. This study was reviewed by the University of Utah’s Institutional Review Board (IRB_00175087) and found to be exempt from further oversight.

## Results

Comparison of the results from CPOE/EHR Evaluation Tool between the MKB vendor’s fully enabled product to the live implementation at a hospital site revealed differences between the overall score and within the order-checking categories. The overall score for the fully enabled, factory settings (in vitro) version of the medication knowledge base was 94%. In 2018, the mean overall score in 2018 was 65.7% (Table 1). Regarding order category scores, most of the mean scores were lower than MKB vendor’s, except for the drug route category, where the MKB vendor scored a 50%, while the mean order category score was 95.3%. However, across all order categories, hospitals achieved a wide range of scores (0%-100%).

**Table 1.** Comparison of CPOE/EHR Evaluation Tool scores across order categories for the medication knowledge base and mean overall performance across all hospitals using the MKB vendor in 2018.

Order category	MKB vendor (in vitro) (%)	Mean scores (2018) (%)	Range of scores (2018) (%)
Drug age	100	22	0-100
Drug allergy	100	96.6	0-100
Drug diagnosis	100	36.1	0-100
Drug dose (daily)	100	80.4	0-100
Drug dose (single)	100	80.6	0-100
Drug route	50	95.3	0-100
Drug-drug interaction	100	78.0	0-100
Therapeutic duplication	100	75.0	0-100
**Overall score**	**94**	**65.7**	**24.2-100**
Fatal orders	100	81.2	0-100

Abbreviations: CPOE/EHR, Computerized Physician Order Entry/electronic health record; MKB, medication knowledge base. Bold values indicate the composite scores (overall score and fatal orders), shown to distinguish them from the individual order category scores.

### Results from the national evaluation of vendor hospitals from 2017 to 2019

#### Hospital characteristics

From 2017 to 2019, 358 hospitals used the MKB vendor’s product to take the CPOE/EHR Evaluation Tool. Most hospitals belonged to a healthcare system (84.1%) and were nonprofit organizations (77.9%) (Table 2). In terms of hospital size, most hospitals (53.7%) were medium-sized (between 100 and 399 beds), while others were small (less than 100 beds) (27%) or large-sized (greater than 400 beds) (19.3%). In terms of location, most hospitals were in urban areas (93.2%), while far fewer were in rural areas (6.8%). Regarding teaching hospital status, most hospitals were not teaching hospitals (90%). Lastly, for EHR vendors, most hospitals used EHR Vendor A (76.8%), followed by EHR Vendor B (10.6%), EHR Vendor C (8.7%), and then EHR Vendor D (3.9%).

**Table 2.** Hospital demographics from national sample of MKB vendor hospitals from 2017 to 2019 (*N* = 358).

Hospital characteristic	*n* (%)
**Healthcare system membership**	
System member hospital	301 (84.1)
Non-system member hospital	57 (15.9)
**Ownership**	
Government-owned	36 (10.9)
Nonprofit	258 (77.9)
For profit	37 (11.2)
**Hospital size**	
Small (less than 100 beds)	95 (27.0)
Medium (between 100 and 399 beds)	189 (53.7)
Large (greater than 400 beds)	68 (19.3)
**Location**	
Urban	316 (93.2)
Rural	23 (6.8)
**Teaching hospital**	
Yes	33 (10.0)
No	298 (90.0)
**EHR vendor**	
A	275 (76.8)
B	38 (10.6)
C	31 (8.7)
D	14 (3.9)

Note that for some of these hospital characteristics, the denominators varied depending on the availability of data from the American Hospital Association’s Annual Survey.

Abbreviations: EHR, electronic health record; MKB, medication knowledge base.

#### Overall performance

**Figure 1.**
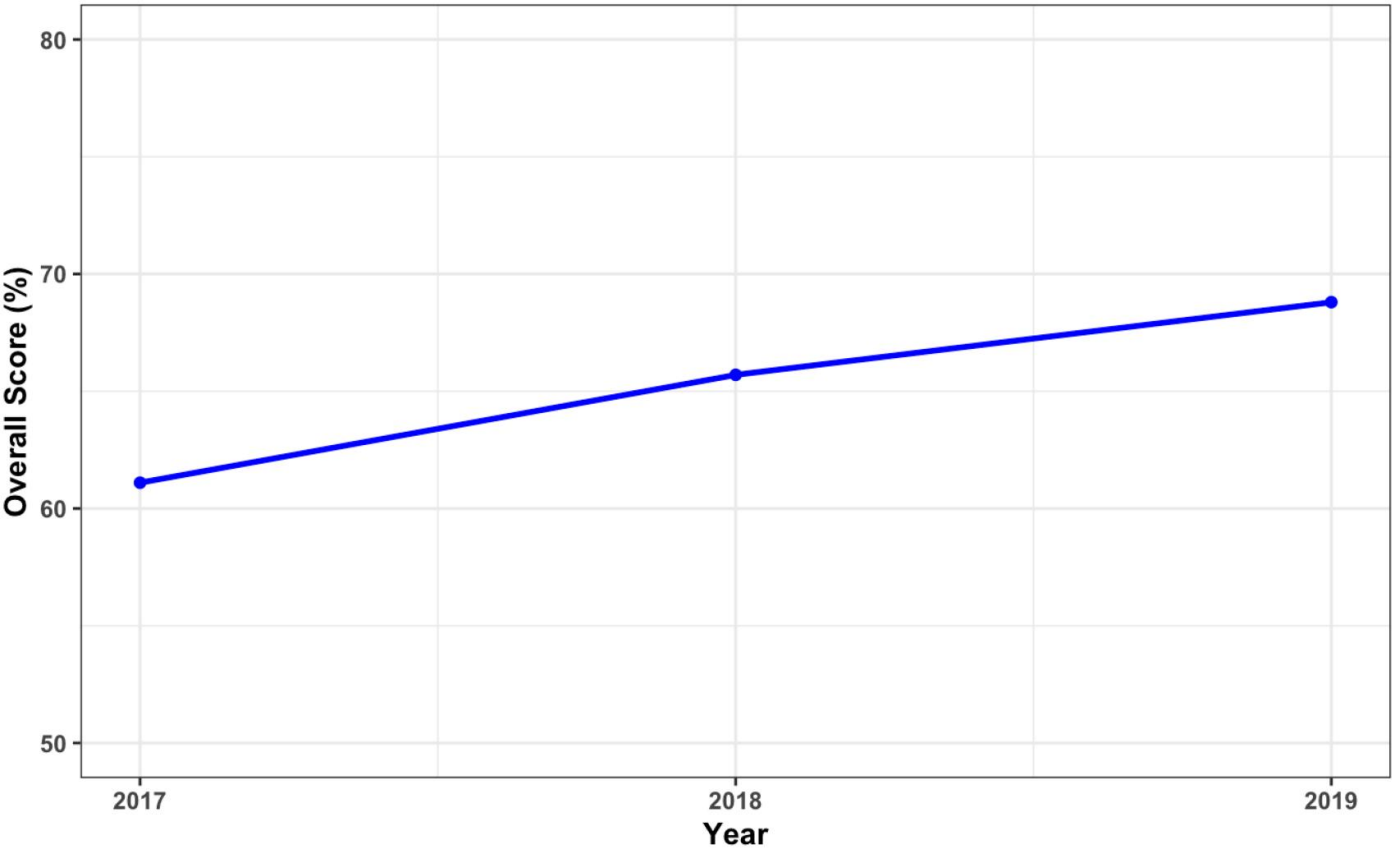
The mean overall scores of hospitals using the medication knowledge base vendor product from 2017 to 2019.

**Figure 2.**
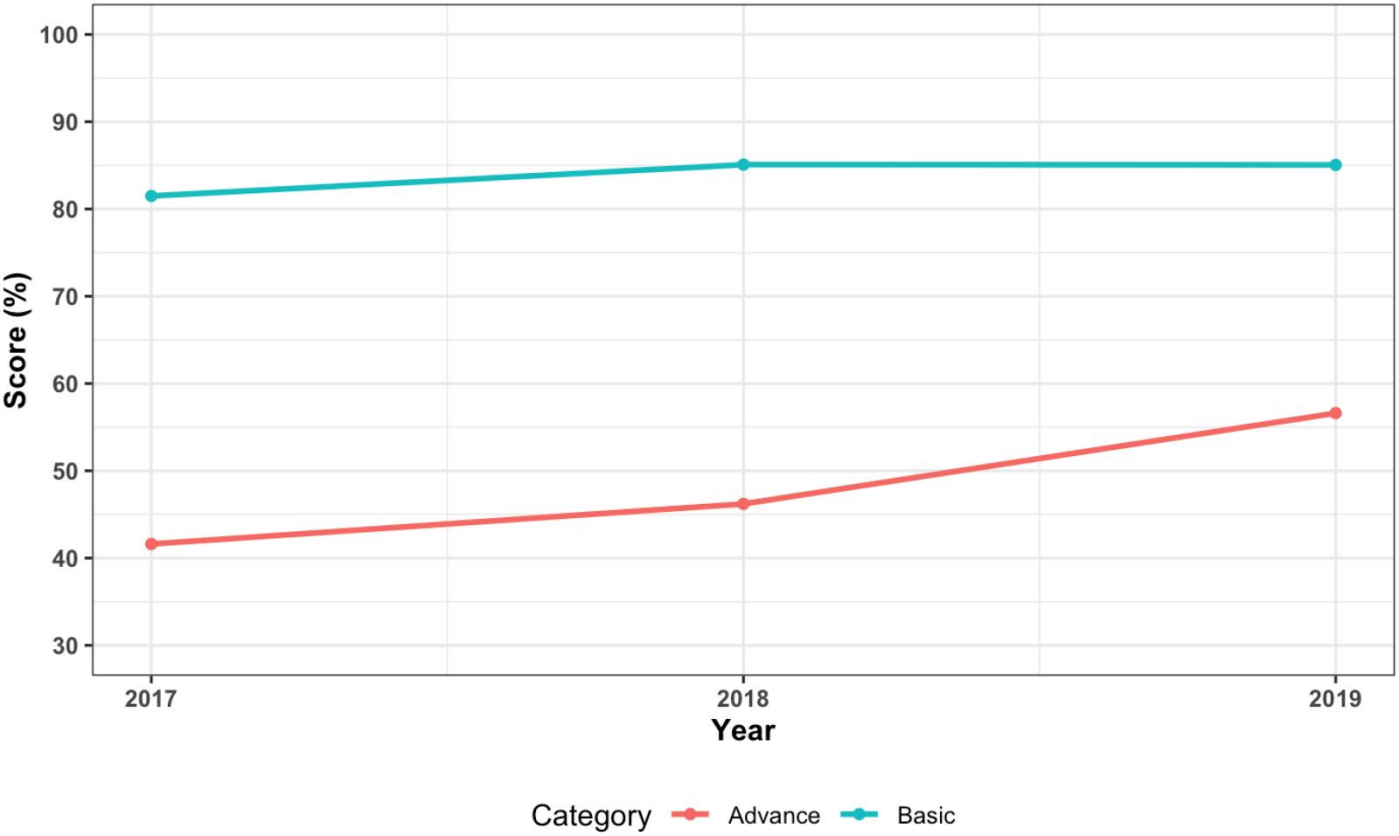
Mean scores for basic and advanced decision support areas from 2017 to 2019. Basic decision support areas included the drug-allergy, drug-route, drug dose (single), therapeutic duplication, and drug-drug interaction order categories.^7^ Advanced decision support areas included the drug dose (daily), drug-age and drug-diagnosis order categories.^7^

**Figure 3.**
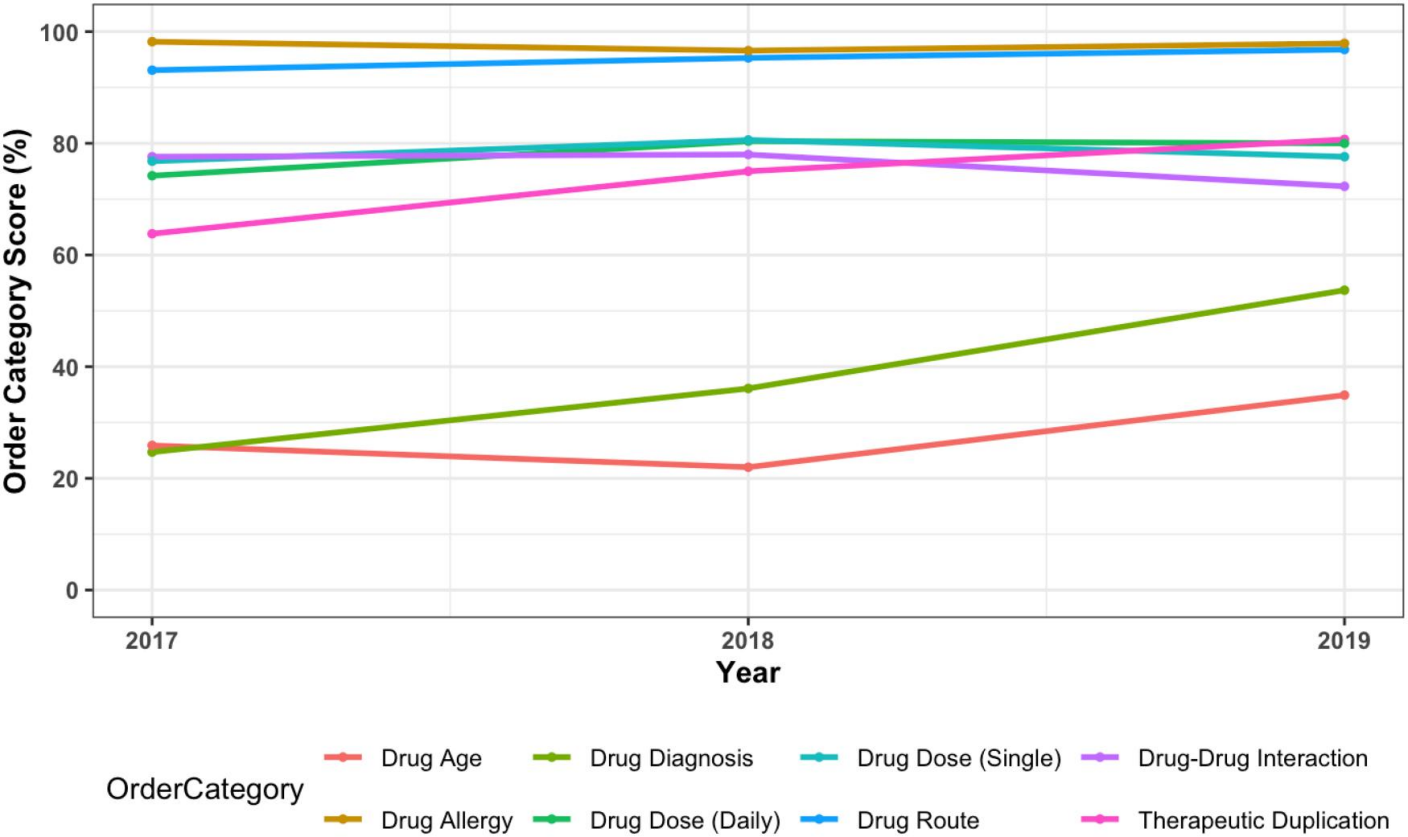
Mean order category scores from 2017 to 2019.

The mean overall score of hospitals using the MKB vendor’s product from 2017 to 2019 were 61.1% (2017), 65.7% (2018), and 68.8% (2019) (Figure 1). Performance in basic and advanced decision support also increased in these years, where for basic CDS categories the mean scores increased from 81.5% to 85%, while in advanced CDS categories, the mean scores increased from 41.6% to 56.6% (Figure 2). For the order-checking categories, performance improved for most of them. These included drug-age, drug-allergy, drug-diagnosis, drug-route, and therapeutic duplication (Figure 3). For drug dose (daily), performance improved from 2017 (74.2%) to 2018 (80.4%) but did not change in 2019 (80.4%). For the drug dose (single) order category, mean scores also increased from 2017 (76.8%) to 2018 (80.6%) but decreased in 2019 (77.6%). A similar pattern was observed in the drug-drug interaction order category, where mean scores increased from 2017 (77.6%) to 2018 (78%) but in 2019 decreased to 72.3%.

#### Performance by EHR vendor

**Figure 4.**
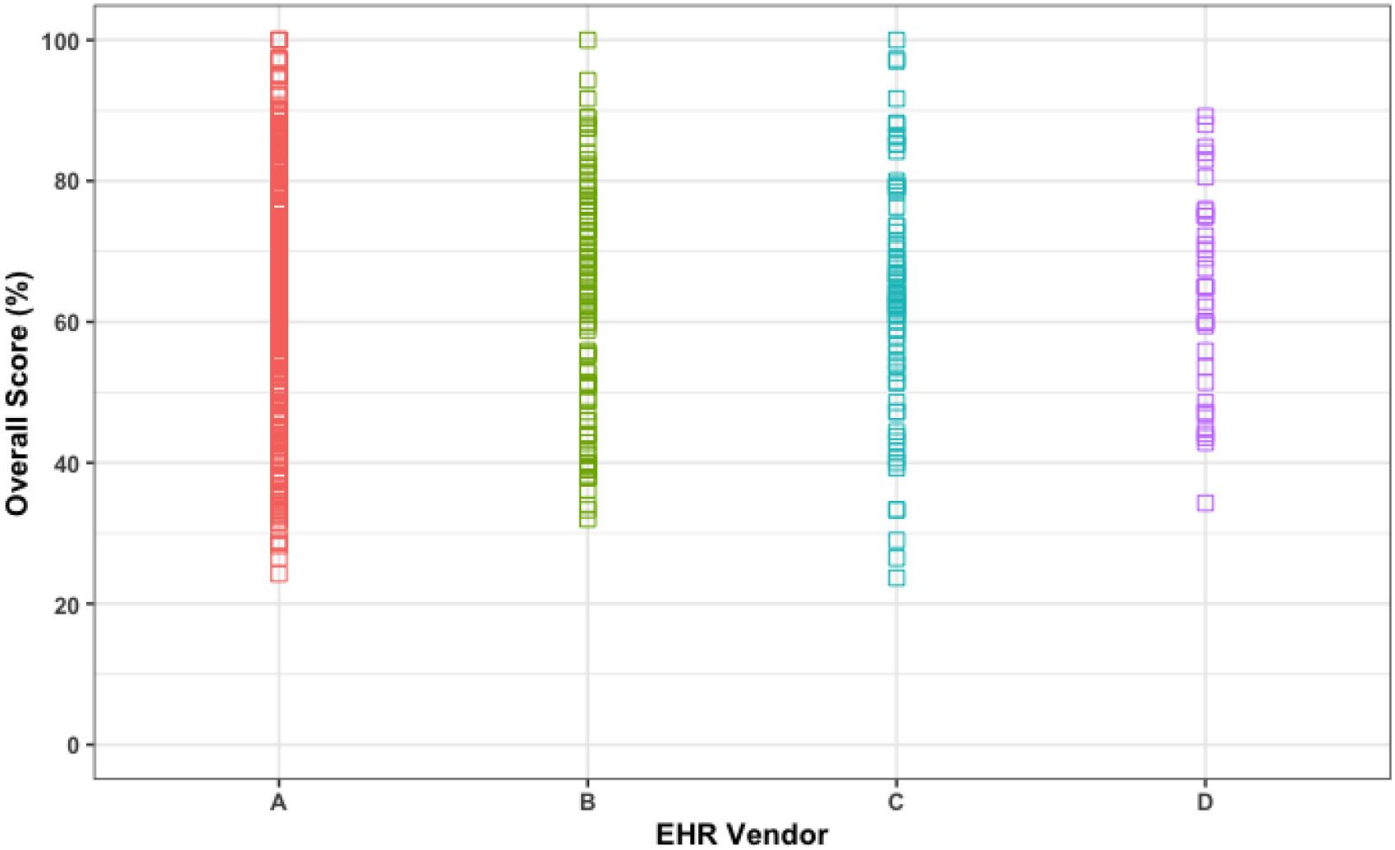
Dot plot showing the range of overall scores by EHR vendor from 2017 to 2019.

Of the 4 EHR vendors assessed in this study, EHR Vendor A had the highest mean overall score across 3 years (65.9%, SD = 14.5%) and overall scores ranged from 24.2% to 100% (Figure 4). For EHR Vendor D, the mean overall score was 64.2% (SD = 14.2%), and the individual hospital scores ranged from 34.3% to 89.2%. This was the only EHR vendor for which no hospital scored 100% within 3 years. Next, for EHR Vendor C, the mean overall score was 63.7% (SD = 16.8%), and individual hospital scores ranged from 23.7% to 100% (Figure 4). This was the largest range (76.3 percentage points) in overall scores among all the vendors. EHR Vendor B’s mean overall score was 62.6% (SD = 16.7), and their individual scores ranged from 32% to 100%.

**Figure 5.**
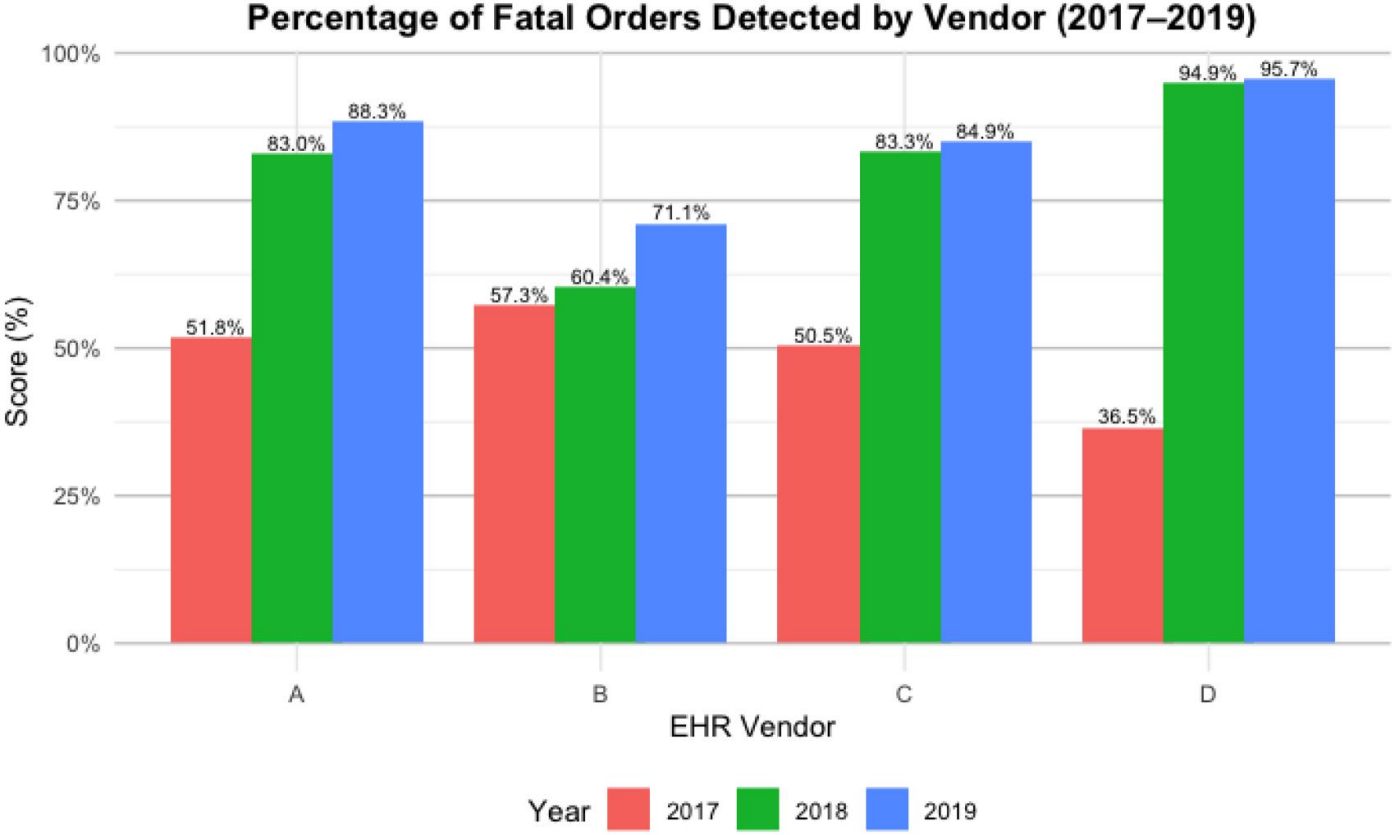
The percentage of fatal orders that were detected within each EHR vendor from 2017 to 2019.

Regarding the fatal orders, performance in this subcategory improved from 2017 to 2019 across all EHR vendors (Figure 5). In 2017, hospitals using EHR Vendor D alerted on the least number of fatal orders (36.5%), but in 2019 improved to 95.7%. For hospitals using EHR Vendor B, their fatal order performance only improved from 57.3% in 2017 to 71.1% in 2019. For EHR vendors A and C, hospitals also improved from approximately 50% in 2017 to over 80% in 2019.

Next, the multiple linear regression model (Table 3) revealed that when controlling for hospital characteristics, EHR Vendor B hospitals scored 3.6 percentage points lower (*P* = .01) than EHR Vendor A hospitals. Electronic health record Vendor C hospitals’ lower performance compared to EHR Vendor A hospitals was not significant (*P* = .053). However, our model explained only 3.7% of the variation in overall scores (*R*^2^=0.037). To determine how much of this variation was due to EHR vendor, we ran the model again without EHR vendor as an independent variable. The adjusted *R*^2^ for this model was 0.036. As a result, the partial correlation between these models was 0.001, or 0.1%. In turn, this indicates EHR vendors only accounted for approximately 0.1% of the variation in overall scores when controlling for observable hospital characteristics.

**Table 3.** Results from the multiple linear regression model with EHR vendor as the primary independent variable, with hospital characteristics as the covariates.

Hospital characteristic	Coefficient	Standard error	*P*-value	[95% CI]
**EHR vendor**				
A	Reference			
B	−3.57	0.73	.01	[−5.89 to 1.25]
C	−2.03	0.65	053	[−4.11 to 0.451]
D	−0.87	0.92	.41	[−3.79 to 2.05]
**Healthcare system membership**	1.32	2.3	.61	[−6.02 to 8.65]
**Ownership**	0.97	1.21	.48	[−2.87 to 4.81]
**Hospital size**	0.67	1.0	.55	[−2.5 to 3.83]
**Location**	−0.18	0.98	.87	[−3.30 to 2.94]
**Teaching hospital**	1.08	1.0	.36	[−2.09 to 4.25]
Adjusted *R*^2^ = 0.037				
Adjusted *R*^2^ with EHR vendor removed = 0.036				

Abbreviation: EHR, electronic health record.

## Discussion

In this study, we used the results from the Leapfrog Group’s CPOE/EHR Evaluation Tool to compare the performance of a MKB vendor’s fully enabled, factory settings product before implementation at a hospital (in vitro) to actual operational implementations in a national sample of hospitals (in vivo) using the same MKB vendor as their medication reference database from the years 2017 to 2019. To our knowledge, this is the first study evaluating the performance of a MKB vendor, rather than the EHR vendor, using the CPOE/EHR Evaluation Tool. The results of this study revealed variations in the implementation and configuration of the MKB vendor’s product across all EHR vendors at the hospital level, and that basic decision support features were more widely implemented compared to advanced decision support features.

The in vitro MKB vendor’s fully enabled product achieved an overall score of 94%, while the mean score for hospitals using the MKB vendor in 2018 was 65.7% (Table 1). In all order categories except for drug route, the MKB vendor had higher performance. During this evaluation, certain unlikely scenarios for drug-route screening were revealed that were previously outside of the MKB vendor’s editorial policy. In response to this, enhancements are now being made to account for all potential scenarios. The overall scores for the hospitals ranged from 24.2% to 100%, indicating significant variability in how hospitals implemented their EHR and MKB systems (Table 1). On top of this, in all the order checking categories, including the fatal orders, the percentage scores ranged from 0% to 100%, indicating that some hospitals had certain types of CDS turned off or not functioning as designed. These results are consistent with our previous work, which has shown a large variation between EHR vendor (not MKB vendor) meaningful use certification performance (in vitro) and the performance among operational systems at hospitals (in vivo).^11^ This is expected as hospitals highly customize EHR vendor products during implementation and the same is true for their medication safety database products. Examples from prior studies include customizing the specific International Classification of Disease (ICD)-10 codes for which drug diagnosis alerts trigger in response to,^16^ and reclassifying the severity of drug-drug interactions from severe to moderate to further refine the delivery of drug-drug interaction alerts.^17^ Similar to EHR vendors, MKB vendors have learned that hospitals insist on the ability to customize their products and thus they deliver that capability. Examples from the MKB vendor include adjusting the minimum severity, minimum documentation level, and minimum management code for which drug interaction alerts are displayed, adjusting the dosing threshold (eg, the percentage above the recommended dose for a medication) for drug dosing alerts, turning on or off certain drug classes for duplicate therapy warnings, and for drug allergy warnings, hospitals can decide whether to include warnings for the individual drug, the drug class, or cross-sensitive classes.

We found that performance improved from 2017 to 2019 across a broad cohort of hospitals that used the MKB vendor’s product. This is consistent with the improvement in overall test scores since the test was first released in 2009, which we believe is related to quality improvement and optimization efforts by both the EHR vendors and hospitals in response to performance in the CPOE/EHR Evaluation Tool.^12^ We also found that hospitals did well on basic decision support category scores, and did not perform as well, in advanced decision support categories, which is also consistent with previous studies.^11^^,^^12^ In particular, the drug-age and drug-diagnosis order categories seem to be an ongoing challenge for hospitals to improve upon even though these categories have vendor product capability and there is evidence that some hospitals do very well on these categories.^11^^,^^12^ Of note, hospitals have challenges with decision support designed to avoid the use of fetal toxic medications. Although MKB vendors (including the one evaluated in this study) have this capability, most hospitals have a difficult time recoding and maintaining accurate pregnancy status in the problem list such that alerts can fire on high-risk drugs for fetuses. In addition, for the drug age category, the tool only assesses for medication-related geriatric alerts. Since dosing in this population is more complex,^18^ having decision support in this area could aid in preventing potential adverse events.^19^ The results of our national evaluation also indicated large variations in the implementation of the MKB vendor’s product across EHR vendors, where the range of overall scores ranged from at least 20%, all the way to 100% (Figure 4). These results are in parallel with a previous evaluation of the tool in 2010 by Metzger et al.,^9^ where similar variations in CPOE/EHR Evaluation Tool performance within hospitals using the same EHR vendor were observed. Indeed, EHR vendor contribution to the variation in our model only accounted for 0.1% of the variation, even when controlling for observable hospital characteristics like healthcare system membership and hospital ownership. This finding is similar to a previous national evaluation of the CPOE/EHR Evaluation Tool with several EHR vendors and medication knowledge bases where the results revealed that EHR vendor choice explained only 9.9% of the variation across 9 years of data.^11^ The decline in how much variation was explained by vendor choice may be related to ongoing optimization of all vendor products at both the vendor and hospital level after the initial implementation, which may explain the product’s test scores over time.

For our model, we initially thought that hospitals that are part of a healthcare system would have higher performance since they usually have more robust health IT departments. However, the results revealed that there was no association, suggesting that there are clearly other factors accounting for the other 96.3% of variation in overall scores. This may be because implementation of EHR systems is a sociotechnical process,^20^ and thus balancing patient safety features and alert fatigue is a complex process with which many healthcare systems continue to struggle. On top of this, hospital customizations of EHR systems and the associated medication database can occur at every level, ranging from the healthcare system as a whole, individual departments, and the end-user. While these customizations are important features within EHR systems, ensuring that there are necessary safeguards in place to prevent serious medication errors is crucial.

Alert precision is likely to improve over time as more alerts are developed that behave differently depending on the specific context (patient, user, location, etc.). In addition, we have observed hospitals using nonalert-based approaches to improve medication safety. Specifically, some hospitals use un-interruptive hard stops to prevent medications from being ordered by the wrong route or dose. Hospitals accomplish this by limiting the dosage or frequency of administration for certain medications to prevent overdoses, and by removing certain drug routes from the order catalogue to prevent medications from being administered by an inappropriate route. An example of this from the tool, is for vincristine, where some hospitals only allow licensed prescribers to order it as an intravenous injection or infusion to prevent any adverse events from erroneously ordering vincristine as an intrathecal injection. In our experience with the CPOE/EHR Evaluation Tool, this is a common approach that many hospitals use. This approach is already decreasing alert fatigue and will continue to evolve.

With these results, it is important to note that the generalizability of these results may be affected by advances to EHR and CDS integrations in recent years. One such advancement is incorporating more context- and patient-specific information into the delivery of alerts, which has led to increased alert acceptance.^21^^,^^22^ In relation to the CPOE/EHR Evaluation Tool, since 2019, the content of the tool has not gone through significant changes, but the organization of the content has. However, even with these changes, similar patterns around the variation in performance of hospitals with the same EHR configurations have been observed. Indeed, in a preliminary study of hospitals that are part of the same healthcare system with the same EHR configuration (same EHR and MKB Vendor) they showed similar variability in overall scores using the 2024 version of the CPOE/EHR Evaluation Tool.^23^ Indeed, in one healthcare system with 17 hospitals, overall scores ranged from 69% to 93%, while another healthcare system with 16 hospitals had overall scores ranging from 26% to 89%. Note, in these 2 healthcare systems, they used the same EHR and MKB vendor.^23^ These data parallel with the findings of this study, in that variation was observed not only across health systems with the same EHR but also among hospitals within the same healthcare system. In turn, this further emphasizes how site-level implementations of EHR and MKB vendor products can affect the performance of CDS tools.

We believe different approaches to implementation and configuration explain the difference between this vendor’s test performance in vitro versus actual implemented hospital scores (in vivo). These approaches might also explain the variation we have previously observed between EHR vendors certification status and the actual hospital performance on the test.^11^ Many have believed that with rule-based decision support in EHRs, there would not be much variation in clinical decision support for medication ordering as seen in certified products and those products in operational use. We have observed over 15 years of testing that there is and remains a lot of variation in medication decision support between Meaningful Use certified products and those products in actual clinical use. Recent testing of EHR systems with artificial intelligence (AI) applications in the decision support arena suggest that there may be even more variation in AI-enabled clinical decision support for medications. Specifically, as hospitals continue to customize these applications, they must also deal with local data differences which will alter the AI system’s performance as well. In turn, the AI system’s performance can drift over time due to these minor changes in data input. Finally, these AI systems will learn over time and alter their performance as part of this learning process. This will be critical as AI rapidly evolves traditional rule-based decision support systems and will have significant implications for certification of AI systems in vitro as proposed for health AI assurance labs.^24^ Local testing, evaluation, and continuous monitoring will be essential for safe use of AI systems in clinical care.^25^^,^^26^

In summary, we believe all hospitals should take the Leapfrog CPOE/EHR Evaluation Tool at least once a year and be required to report their results. Indeed, in a previous longitudinal study,^12^ hospitals which took the test multiple times performed better in subsequent years compared to hospitals taking the test for the first time. In turn, this suggests that consistent voluntary self-assessments can aid in progressive improvements to new and existing CDS tools. In addition, the CPOE/EHR Evaluation Tool is part of the Safety Assurance Factors for EHR Resilience (SAFER) guides, which provides recommendations to healthcare institutions for self-assessment of their EHR systems.^27^ In 2022, the Centers for Medicare and Medicaid Services started requiring healthcare systems to attest annually to reviewing the SAFER guides.^28^ Self-assessment evaluations like the CPOE/EHR Evaluation Tool are therefore valuable tools for healthcare systems to assess continually the performance of their decision supports systems in preventing adverse events. Thus, as new AI applications in medication ordering are implemented at hospitals, we think these applications should also be tested with a similar approach to the Leapfrog CPOE/EHR Evaluation Tool at least annually and perhaps more frequently.

### Limitations

Our national evaluation of the MKB vendor product was only performed with hospitals which took the CPOE/EHR Evaluation Tool from 2017 to 2019. This sample of hospitals did not include all hospitals which used the MKB vendor product. Next, the content in the CPOE/EHR Evaluation Tool is not inclusive of all types of medication errors that hospitals encounter. However, in developing the content for this tool, experts derived the medication test orders from real-world cases. For this study, we did not link our test results to patient outcomes, we only assessed the functionality of the EHR systems to detect and prevent adverse drug ordering events. However, a previous study by Leung et al.^10^ found a 43% relative reduction in the rate of preventable adverse drug events (ADEs) for every 5% increase in the scores in the CPOE/EHR Evaluation Tool. Next, the trends in overall performance presented in this study may not be completely representative of current performance of the tool. However, performance in the tool has consistently improved overall and across the different order categories. Lastly, while the CPOE/EHR Evaluation Tool asked hospitals about their primary EHR and knowledge base vendors, there may be some hospitals for which other ancillary knowledge base systems were used for certain types of alerts. Data on these other systems were unavailable for this study.

## Conclusion

In vitro, the MKB vendor’s fully enabled product achieved a 94% on the Leapfrog CPOE/EHR Evaluation Tool, but the mean in vivo (real world) performance among 358 hospitals ranged from 61% to 69% over the 2017-2019 period. There was widespread variation of scores for each of the 4 EHR vendors studied. Our linear regression model found that the choice of EHR vendor had almost no effect on the overall scores. Therefore, the choice of EHR vendor is not as important as the choices made in configuring the MKB vendor’s product within a specific EHR as it relates to scores on the CPOE/EHR Evaluation Tool. For patient safety to improve, healthcare organizations will have to turn on more types of CDS. For this increase in medication alerts to be tolerable for clinicians, MKB and EHR vendors will have to continue to work together to increase the specificity of their alerts.

## Data Availability

The data used in study cannot be publicly shared for privacy and proprietary reasons.
